# Role of digital health in the personalized management of heart failure due to Fontan failure: a case report

**DOI:** 10.1093/ehjcr/ytag733

**Published:** 2026-10-07

**Authors:** Elena Cavarretta, Maria Teresa Quarta, Gianfranco Butera, Antonio Amodeo, Rachele Adorisio

**Affiliations:** Heart Failure, Transplant and Mechanical Assist Device Unit, Bambino Gesù Children's Hospital, IRCCS, Piazza Sant'Onofrio, 4, 00165 Rome, Italy; Department of Medical-Surgical Sciences and Biotechnologies, Sapienza University of Rome, 04100 Latina, Italy; Department of Pediatrics, University ‘G. D'Annunzio’ of Chieti-Pescara, 66100 Chieti, Italy; Interventional Cardiology Unit, Pediatric Cardiology, Cardiac Surgery and Heart, Lung Transplantation, Bambino Gesù Children's Hospital, IRCCS, 00165 Rome, Italy; Heart Failure, Transplant and Mechanical Assist Device Unit, Bambino Gesù Children's Hospital, IRCCS, Piazza Sant'Onofrio, 4, 00165 Rome, Italy; Department of Cardiothoracic Surgery, Stanford University School of Medicine, Stanford, CA 94305, USA; Heart Failure, Transplant and Mechanical Assist Device Unit, Bambino Gesù Children's Hospital, IRCCS, Piazza Sant'Onofrio, 4, 00165 Rome, Italy

**Keywords:** Telemonitoring, Digital health, Congenital heart disease, Pediatric heart failure, Case report

## Abstract

**Background:**

Heart failure (HF) represents a major cause of morbidity and mortality in patients with Fontan circulation. Chronic venous congestion, impaired preload, and limited cardiac output reserve make clinical assessment and intermittent invasive evaluations insufficient to capture early haemodynamic deterioration. Pharmacological treatment lack of robust evidence in this subgroup of patients. In this challenging setting, implantable invasive haemodynamic monitoring systems may provide continuous, real-life information capable of guiding personalized HF management.

**Case summary:**

A 26-year-old Fontan male developed protein losing enteropathy and recurrent hospitalizations for decompensated heart failure. Therefore, the patient was candidate to heart transplantation. Over time, episodes of acute HF increased with an escalating requirement of dosage of diuretics. At 22 years of age, another episode of acute decompensated HF prompted a catheter-based intervention with Fontan conduit re-dilatation and re-fenestration with an Atrial Flow Regulator (AFR) implant, and a pulmonary artery pressure monitoring system (CardioMEMS™) was deployed to telemonitor pulmonary artery pressures and adjust the diuretic therapy accordingly. Up to date, 4 years from the intervention, the use of this remote monitoring system was feasible and effective in tailoring medical therapy to prevent further hospitalizations for acute HF. The patient is in clinical follow-up with regular controls.

**Discussion:**

Systems allowing continuous or repeated assessment of haemodynamic status in real-life conditions may support personalized treatment adjustments, improve symptom control, and potentially reduce HF-related hospitalizations in patients with failing Fontan circulation.

Learning points

- Heart failure due to Fontan failure is a complex syndrome, due to heterogeneity of the patients, the lack of paediatric scientific evidence and the recurrent hospitalizations.
- Remote haemodynamic monitoring trends obtained via an implantable pulmonary artery pressure sensor were feasible and of potential clinical utility to guide individualized adjustment of diuretic therapy.
- The favourable clinical course may be due to the synergistic effect of the haemodynamic procedure performed concurrently with implantation of the pulmonary artery pressure sensor.

## Introduction

Fontan palliation has markedly improved survival in patients with functionally univentricular hearts, resulting in a steadily increasing population of children and adults living with a Fontan circulation. Despite these advances, heart failure (HF) remains a major determinant of morbidity and mortality in this population.^1,2^ HF associated with Fontan physiology is a distinct clinical entity, driven by unique pathophysiological mechanisms that include chronically elevated central venous pressure, reduced cardiac output, impaired ventricular preload, and a severely limited ability to increase cardiac output during exercise or physiological stress. These haemodynamic constraints, frequently exacerbated by residual anatomical obstructions, ventricular dysfunction, arrhythmias, and lymphatic abnormalities, promote progressive multiorgan involvement, with common manifestations including hepatic disease, renal dysfunction, and protein-losing enteropathy.^3^ As a result, Fontan patients often experience a gradual clinical decline punctuated by episodes of acute decompensation. Risk stratification and haemodynamic assessment are integral components of routine clinical follow-up in this setting.^4^ However, despite an improved understanding of the clinical phenotype and natural history of Fontan-related HF, evidence-based medical treatment strategies remain poorly defined and are largely extrapolated from non-congenital HF populations.^5^ In this context, advanced monitoring systems capable of providing continuous or repeated invasive haemodynamic assessment may represent a valuable tool to guide individualized therapeutic adjustments, enabling more proactive and tailored management of HF in patients with a failing Fontan circulation.

## Summary figure

**Figure:**
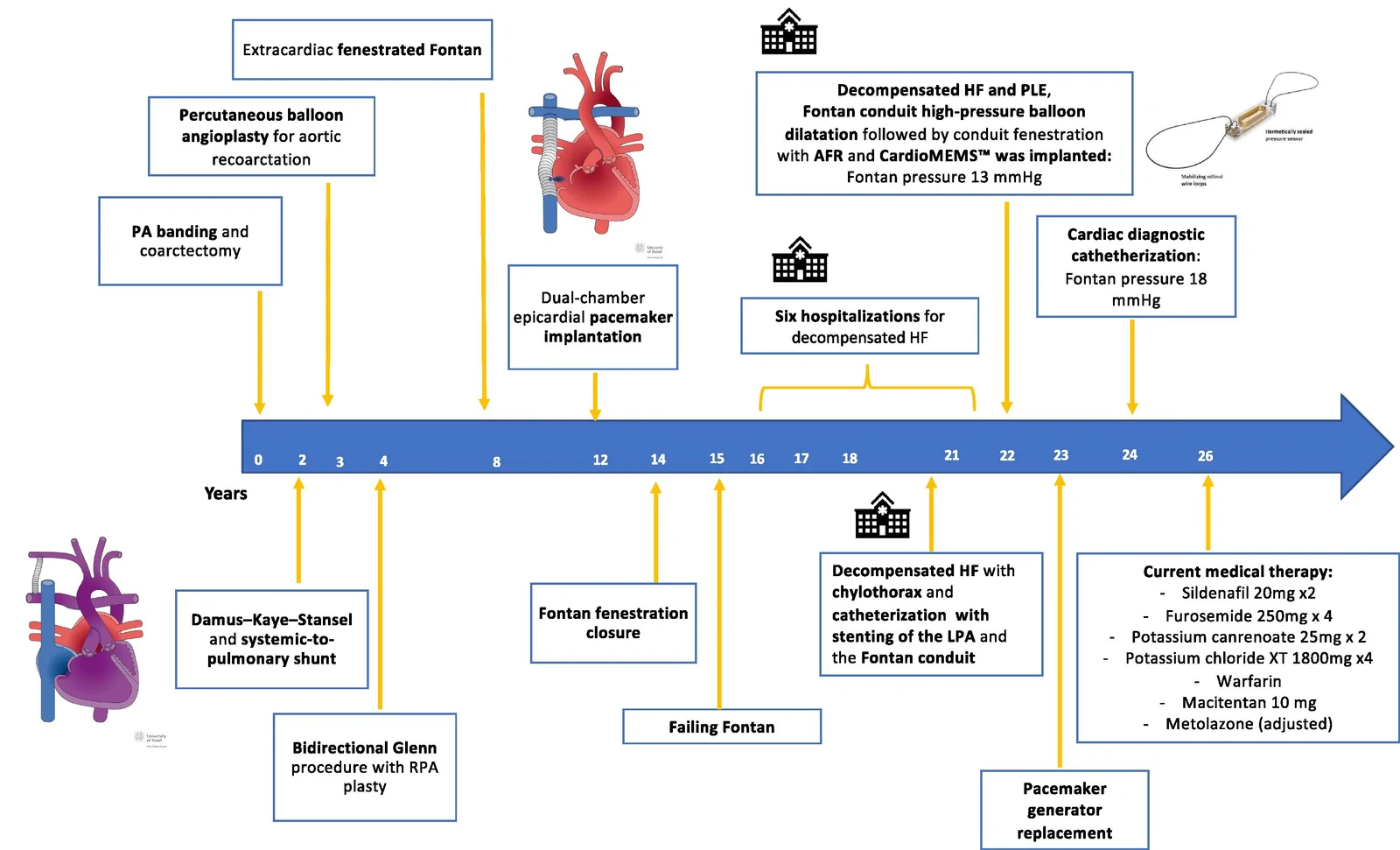
Timeline of staged cardiac interventions from pulmonary artery banding at birth to extracardiac fenestrated Fontan at 8 years, fenestration closure at 14 years, and failing Fontan at 15 years with six hospitalizations for decompensated heart failure between 16 and 21 years of age. At 22 years of age, another episode of acute decompensated HF prompted a catheter-based intervention with Fontan conduit re-dilatation and re-fenestration with an Atrial Flow Regulator (AFR) implant and pulmonary artery pressure monitoring system (CardioMEMS™, Atlanta, Georgia, US) implantation. Abbreviations: AFR, atrial flow regulator; HF, heart failure; LPA, left pulmonary artery; PA, pulmonary arteries; PLE, protein-losing enteropathy; RPA, right pulmonary artery. Cardiac morphology images provided by http://www.chd-diagrams.com. Image CardioMEMS courtesy of CardioMEMS^TM^, Atlanta, Georgia, US. Short alternative text description

## Case presentation

A 26-year-old male with complex congenital heart disease, including double-inlet left ventricle, subaortic stenosis, and aortic coarctation, associated with unilateral renal agenesis, underwent staged surgical palliation towards a Fontan circulation. Initial management included pulmonary artery banding and coarctectomy at birth, followed at 2 years of age by a Damus–Kaye–Stansel procedure with a systemic-to-pulmonary shunt and aortic arch reconstruction, complicated by a cerebral embolic event resulting in left hemiparesis. Subsequent interventions included balloon angioplasty for aortic recoarctation, bidirectional Glenn procedure with right pulmonary artery plasty, and Fontan completion at 8 years of age using a fenestrated extracardiac conduit (*Summary figure*).

During adolescence, the patient developed progressive Fontan failure. A dual chamber epicardial pacemaker was implanted for sinus node dysfunction (*Figure 1*, panel A), and Fontan fenestration closure was performed at 14 years of age, with a conduit pressure of 16 mmHg. One year later, he presented with generalized oedema, cyanosis, reduced exercise tolerance, mildly impaired ventricular function, and protein-losing enteropathy, leading to listing for heart transplantation. Over the following three years, however, the patient experienced recurrent hospitalizations for acute decompensated heart failure requiring escalating doses of intravenous diuretics and inotropic support, as well as prolonged admissions complicated by gastrointestinal bleeding and pleural effusions.

**Figure 1:**
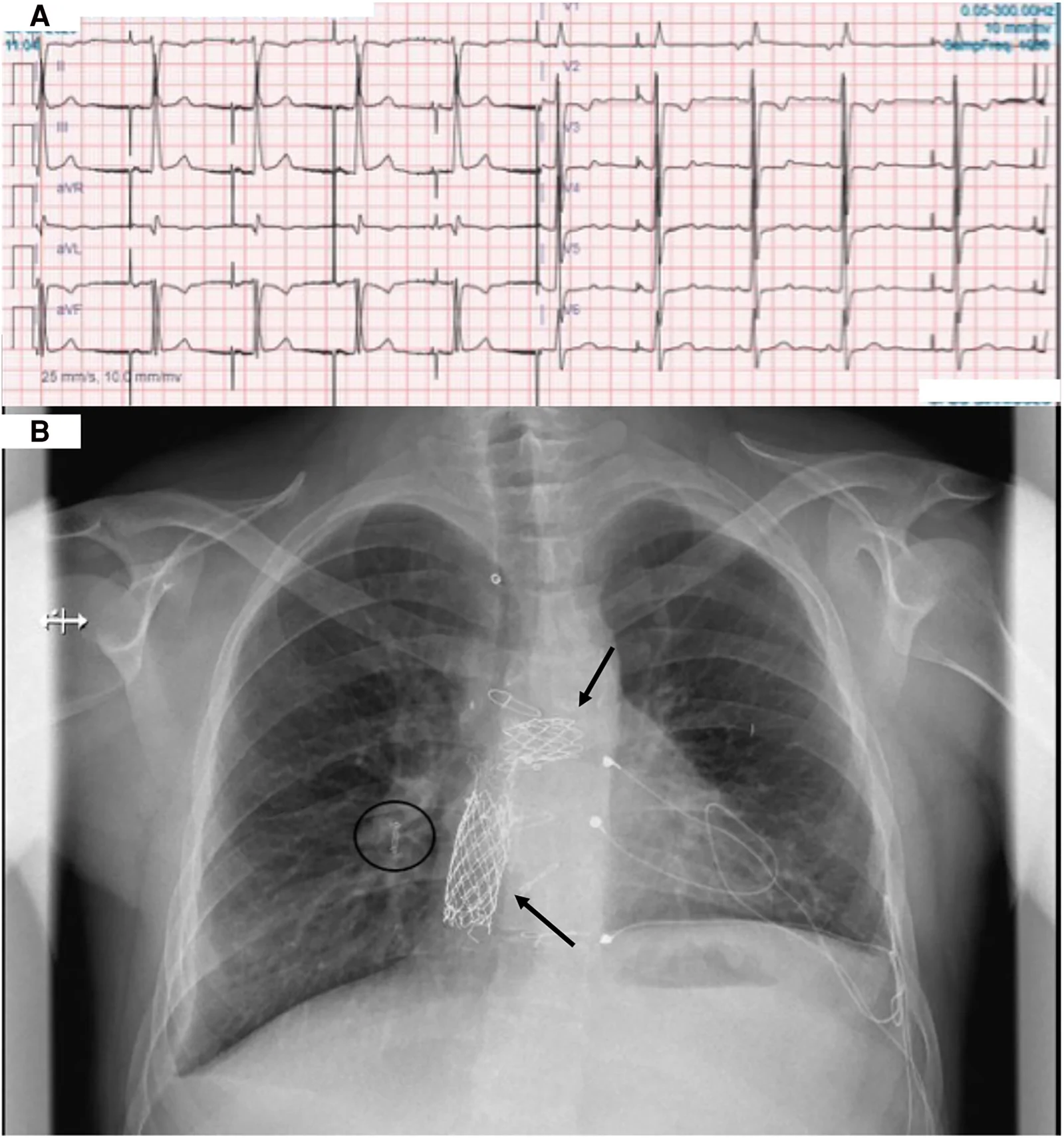
(*A*) Admission 12-lead electrocardiogram showing atrial pacing at 59 beats per minute with spontaneous ventricular conduction, normal intraventricular conduction, R-wave predominance in lead V1. (*B*) Chest radiograph demonstrating the CardioMEMS™ (CardioMEMS^TM^, Atlanta, Georgia, US) device (highlighted by a black circle) in the right pulmonary artery and stents within the Fontan conduit and the left pulmonary artery (black arrows). The pacemaker leads are also present in the right atrium and ventricle.

Despite repeated catheter-based interventions, including pulmonary artery and Fontan conduit stenting, the patient remained in NYHA functional class III–IV with diuretic dependence. Between 16 and 21 years of age, the patient experienced six hospitalizations for acute decompensated HF requiring intravenous diuretic infusions; at 21 years, a haemodynamic procedure with stenting of the left pulmonary artery and the Fontan conduit was performed. At 22 years of age, another episode of acute decompensated HF prompted a catheter-based intervention with Fontan conduit re-dilatation and re-fenestration with an Atrial Flow Regulator (AFR) implant. At the same time, an implantable pulmonary artery pressure monitoring system (CardioMEMS™, Atlanta, Georgia, US) was deployed to guide therapy (*Figure 1*, panel B). Immediately after the successful intervention, CardioMEMS™ monitoring revealed a mean pulmonary artery pressure (PAP) of 13 mmHg. At follow-up, serial monitoring identified several episodes of increased mean PAP >15 mmHg (peak 25 mmHg), which were managed with timely adjustments of oral diuretic therapy, including the addition of metolazone once the loop diuretic furosemide was already administered at the maximum dose (250 mg PO 4 times a day, total daily dose 1000 mg). Because Fontan conduit re-dilatation and re-fenestration were performed concurrently with CardioMEMS™ implantation, the relative contribution of pressure-guided management to the subsequent clinical stabilization cannot be determined independently. Nevertheless, the combined approach—interventional procedure coupled with remote monitoring of mean PAP—proved feasible and of potential clinical utility for guiding tailored diuretic therapy during the subclinical haemodynamic deterioration that followed the procedure. To achieve adequate weight control and mean PAP <15 mmHg, furosemide was carefully uptitrated using high-dose tablets, starting at 250 mg orally per day and increasing by 250 mg at least every 48 h, up to a total of 1000 mg per day. Once this dosage was reached, metolazone was added whenever the mean PAP rose >15 mmHg, assessed on a daily basis, and discontinued once the mean PAP fell <15 mmHg. The complete medical therapy regimen is summarized in *Summary figure*, while *Figure 2* illustrates the rise in mean PAP observed during monitoring after the interventional procedure and its subsequent successful reduction following medical management. Serum and urinary electrolytes, blood urea nitrogen (BUN) and creatinine were monitored routinely on a monthly basis. Over a 4-year follow-up, the patient did not experience further acute HF hospitalizations, remaining clinically stable after the haemodynamic procedure and remote haemodynamic monitoring coupled with tailored medical management. No episodes of electrolyte disturbance or significant creatinine increase (> 20%) were observed during follow-up.

**Figure 2:**
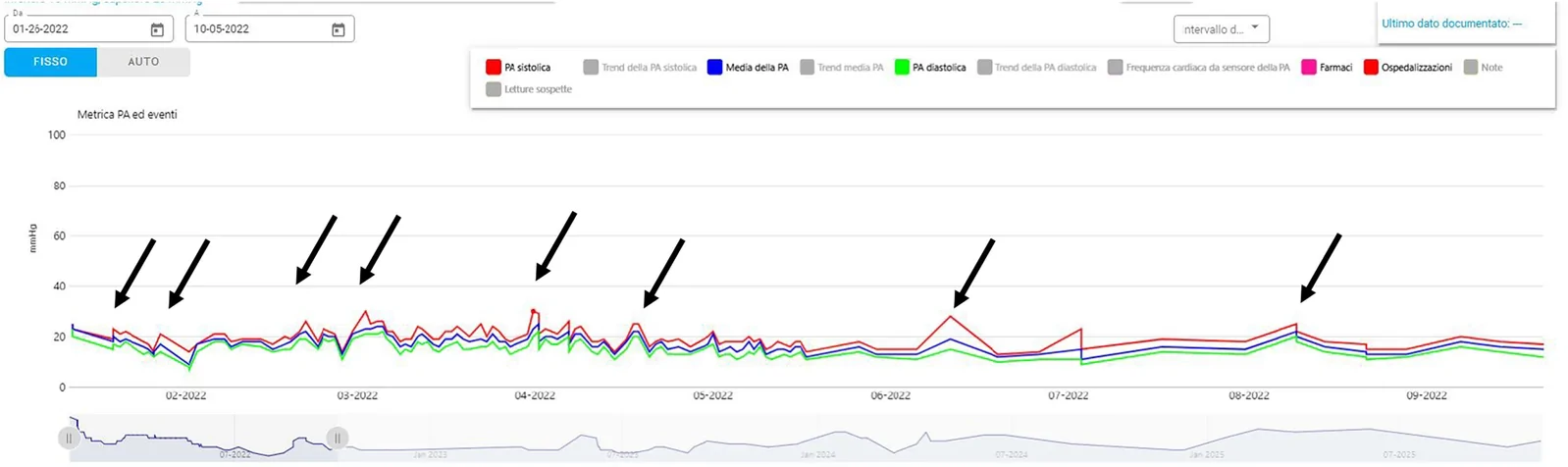
Trend of systolic, diastolic, and mean pulmonary artery pressures (PAP), recorded by the CardioMEMS™ (CardioMEMS^TM^, Atlanta, Georgia, US) system over an 8-month period. The red line represents systolic pulmonary artery pressure, the green line represents diastolic pulmonary artery pressure, and the blue line represents the mean pulmonary artery pressure. Increases in pulmonary pressure trends >15 mmHg (black arrow) were associated with timely adjustments of diuretic therapy with furosemide and metolazone, followed by an evident decrease in PAP after medical management.

## Discussion

Invasive cardiac catheterization remains the gold standard for identifying venous congestion, the onset of chronic venous hypertension, increased pulmonary vascular resistance, and reduced cardiac output in Fontan patients. It is recommended as a class IC indication in the ESC guidelines for cases of unexplained oedema, exercise intolerance, new-onset arrhythmia, cyanosis, and haemoptysis,^6^ and every 10 years as a surveillance test during adolescence and adulthood according to the AHA guidelines.^7^ At a median follow-up of 10 years, independent predictors of mortality include a central venous pressure ≥16 mmHg and mean systemic arterial pressure <60 mmHg.^8^ Monitoring of PAP is therefore pivotal in the lifelong follow-up of Fontan patients. It is worth noting that, in the supine position and in the absence of an anatomic obstruction, central venous pressure (CVP) is equal to PAP and to the pressure in the superior and inferior vena cava and the Fontan conduit.^9^ Recently, the use of an implantable haemodynamic monitoring device (CardioMEMS™, Abbott, USA) placed in the pulmonary artery of patients with Fontan circulation has emerged as a promising tool.^10, 11^ The CardioMEMS™ system allows serial, noninvasive evaluation of PAPs under real-life conditions, unlike cardiac catheterization, during which the patient is fasting, sedated, sometimes under positive-pressure ventilation.^9^ The first paediatric case series^12^ was promising, showing no procedural complications, no subsequent device migration or thrombosis; clinical benefit was more evident in two patients in whom PAPs were underestimated at cardiac catheterization compared to real-life, due to differences in altitude between the hospital and home in one case and sedation and intubation during the procedure in another case. On one hand, the first single-center retrospective study of 17 adult patients with Fontan circulation failed to demonstrate a reduction in HF hospitalization, showing no significant differences in the rate of HF hospitalization per year before and after the device implant (median 1 [IQR 0–1.0] vs. 0.6 [0–2.3]; *P* = 0.268),^9^ probably due to low adherence to device transmission. On the other hand, despite the high burden of HF, evidence supporting the use of conventional guideline-directed medical therapy in Fontan patients remains limited and inconsistent. Pharmacological strategies commonly extrapolated from adult HF—including beta-blockers, angiotensin-converting enzyme inhibitors, mineralocorticoid receptor antagonists, angiotensin receptor–neprilysin inhibitors, and sodium–glucose cotransporter-2 inhibitors—lack robust randomized data in the Fontan population.^5^ Therapies targeting pulmonary vascular resistance are frequently employed, yet their impact on long-term outcomes remains uncertain, resulting in highly individualized and heterogeneous management approaches. The use of diuretics in patients with Fontan failure also remains controversial. Given these limitations, there is a critical need for strategies that enable optimized and timely use of available medical therapies. Systems allowing continuous or repeated assessment of haemodynamic status in real-life conditions may support personalized treatment adjustments, improve symptom control, and potentially reduce HF-related hospitalizations in patients with a failing Fontan circulation.

This case highlights the importance of education and active involvement of CHD patients in digital health and remote monitoring. This patient experienced several HF-related hospitalizations before undergoing the combined approach of conduit re-dilatation and re-fenestration concurrently with CardioMEMS™ implantation; thereafter, the patient showed high adherence to remote monitoring which facilitated tailored medical management. Patients with end-stage CHD-related HF likely represent an optimal population for digital health monitoring, given their high rate of adverse events and impaired survival, their young age and digital literacy, and the usual presence of caregivers. In addition, remote monitoring may be more effective when coupled with personalized, frequent adjustments of medical therapy and matched with active patient engagement in self-care. The opportunities and challenges of digital health monitoring are summarized in ***Table 1***. Digital health holds promise in end-stage HF patients,^13^ particularly in CHD, but interventions should be evaluated for efficacy and efficiency across multiple domains—ranging from clinical outcomes to patient education and self-care, and from privacy and cybersecurity threats to resource allocation.

**Table 1:** Opportunities and challenges in digital health for remote telemonitoring in patients with Fontan failure

Digital Health for Remote Telemonitoring in Patients with Fontan Failure	
Opportunities	Challenges
Remote monitoring in rural areas or in altitude	Data security, privacy and cybersecurity threats
Monitoring in real-life setting	Patient’s adherence
Improvements in patients’ education	Digital literacy
Engagement in self-care	User-friendly platform and dashboard
Improved outcomes	Connectivity disparities
Reduction in unplanned/emergency hospitalization	Variable/undefined reimbursement
Personalized therapy	Barriers to implementation
Patient-centered healthcare	Clinical validation in RCTs

RCT, randomized clinical trials

**Patient perspective** Paediatric patients with HF due to complex CHD experience a significant impact on various aspects of life and reduced quality of life compared to other individuals, due to repeated hospitalizations, surgical interventions, and lifelong clinical follow-up. If systematically used, an implanted haemodynamic monitoring system may be beneficial in a combined approach to help the patient come to terms with his/her medical condition, individualize medical therapy, identify unexplained symptoms, and facilitate communication between the patient, his/her caregivers and the clinicians and allied care professionals.

## Lead author biography


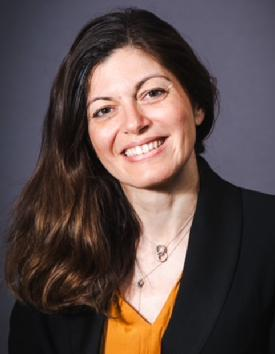
Elena Cavarretta is a MD, PhD and Associate Professor at Sapienza University of Rome, Italy and a scientific consultant for Heart Failure, Transplant and Mechanical Assist Device Unit, Bambino Gesu’ Children’s Hospital, IRCCS Rome. Her scientific interests are related to preventive cardiology, sports cardiology, the paediatric athlete's heart, paediatric cardiomyopathies and exercise prescription in paediatric heart failure. She is a Deputy Editor for European Heart Journal-Case Report (Preventive Cardiology section) and a junior Deputy Editor for European Journal of Preventive Cardiology.

## Data Availability

The data underlying this article will be shared on reasonable request to the corresponding author.
